# Non-Thermal Food Processing Technologies and Polyphenols: LC-MS Evidence for Stability, Transformation, and Functionality

**DOI:** 10.3390/foods15081383

**Published:** 2026-04-16

**Authors:** Chengxuan Li, Cundong Xie, Kashif Ghafoor, Hafiz A. R. Suleria

**Affiliations:** School of Agriculture, Food and Ecosystem Sciences, Faculty of Science, The University of Melbourne, Parkville, VIC 3010, Australia; chengxuan.li.1@student.unimelb.edu.au (C.L.); frankie.xie@student.unimelb.edu.au (C.X.)

**Keywords:** metabolomic fingerprinting, ultrasound, high-pressure processing, pulsed electric fields, cold plasma

## Abstract

Phenolic compounds contribute to the color, flavor, and functionality of foods but are often degraded during conventional heat treatments, prompting interest in non-thermal techniques. Thermal methods produce heat-driven changes that are more directly interpretable, whereas non-thermal methods require compound-resolved interpretation because higher post-treatment signals may reflect release from bound pools rather than true preservation. This review evaluates liquid chromatography–mass spectrometry (LC–MS) evidence on how ultrasound, high-pressure processing, pulsed electric fields, and cold plasma reshape polyphenol fingerprints across food matrices (2021–early 2026). Ultrasound and high-pressure processing preserve constitutive structures while increasing measurable phenolics through cell disruption and bound-pool release. Pulsed electric fields show similar release behavior but may shift toward oxidative losses when electroporation increases enzyme contact or downstream operations amplify degradation. Cold plasma introduces reactive oxygen and nitrogen species, with the clearest LC–MS/MS evidence for oxidation and nitration. In fresh-cut tissues, stress responses elevate phenylpropanoid products. Bulk assays such as total phenolic content (TPC) cannot separate preservation from release or true chemical conversion alone. LC–MS offers the compound-level detail needed to understand how each non-thermal technique changes polyphenol structure and functionality across food matrices.

## 1. Introduction

Phenolic compounds, notably flavonoids including anthocyanins and phenolic acids, are ubiquitous plant secondary metabolites that shape the sensory and functional attributes of food matrices. In this review, the term ‘polyphenols’ is used broadly to refer to major dietary phenolic compounds, including phenolic acids, flavonoids, and tannins. In some beans and tea, these molecules contribute to color, bitterness, and astringency and are key determinants of consumer acceptance and perceived quality [1,2]. Beyond sensory attributes, polyphenols are widely recognized for their potential to reduce the risk of chronic diseases. These include cardiovascular and metabolic disorders, and this effect is primarily mediated through antioxidant and anti-inflammatory mechanisms [3]. At the same time, polyphenols are treated as functional ingredients in processing and storage strategies. When it comes to protein-rich systems, such as meat, they can inhibit lipid and protein oxidation, maintaining safety and extending shelf life [4]. These roles make polyphenols consequential targets of food processing and a recurrent basis for claims related to quality and functionality. This relevance arises not only from the number of polyphenols, but also from their molecular form, stability, and association with the food matrix.

Understanding these processing effects is important for researchers as the same post-treatment increase in signal can arise from preservation, release, or structural conversion, and distinguishing between these requires compound-resolved analytical evidence. It matters equally to food manufacturers because such changes influence color, flavor, functional positioning, process selection, and quality assurance targets. The analytical tools used to assess these effects therefore determine what can be concluded—this point is developed in the sections that follow.

However, some processing methods designed to ensure microbial safety and desirable sensory properties may also adversely affect the chemical integrity of polyphenols. For example, thermal processing methods are essential for microbial control and flavor development. However, in cases of certain thermal treatments such as roasting and boiling, the chemical stability of these bioactive compounds can be compromised [5,6]. This vulnerability is not uniform across polyphenol subclasses, and certain groups are particularly thermolabile. Anthocyanins illustrate why heat cannot be regarded as a neutral processing condition, as elevated temperatures often promote the degradation of thermosensitive molecules and can induce structural destabilization and ring cleavage, leading to color loss and reduced bioactivity [7]. Thermal processing also changes the quality of food in ways that are easy to miss when analytical proof is not direct. Heat treatment speeds up the loss of volatile fragrance components by encouraging reactions that break them down. This changes the sensory profile of the final product [8]. Thermal processing sometimes leads to new chemical risks, including the formation of hazardous compounds such as heterocyclic amines in meat, which polyphenols may help mitigate through radical scavenging or adduct formation [4,9]. Even the seemingly simple question of whether heating lowers phenolics is complicated because measured totals often reflect competing activities. After heating, phenolic content balances free compound breakdown and food matrix release of bound phenolics [10]. Taken together, while essential for safety and product quality, thermal processing alters the compounds that underpin sensory characteristics and potential health relevance.

To mitigate the adverse effects associated with thermal treatments, non-thermal technologies including ultrasound and cold plasma have been applied to preserve the nutritional quality of grains and flours [6]. However, a less well-resolved issue underlies the preservation narrative. Minimizing heat exposure does not equate to preservation of molecular integrity. Although these approaches limit thermal degradation, they are not chemically inert and can still modify the food matrix by changing the physical structure of starch and proteins, thereby forming non-covalent complexes with phenolics and altering their bioavailability and bioaccessibility [6,11]. Here, processing-induced modifications are used in the broad sense and may arise under either thermal or non-thermal conditions; the key point is that matrix restructuring, not heat alone, can alter phenolic availability. In some systems, matrix restructuring promotes the formation of inclusion complexes between polyphenols and starch (V-type amylose complexes), which entrap bioactive molecules and alter release kinetics. Accordingly, even non-thermal processing can modify phenolic availability through physical restructuring and non-covalent interactions [11]. The interpretive challenge that follows is central to this review. After non-thermal treatment, unchanged or higher signals for native phenolics may reflect preservation and/or enhanced extractability, whereas true conversion refers to the formation of different molecular entities and should be interpreted separately. These outcomes are not equivalent when the objective is to understand chemical transformation, bioaccessibility, and functional relevance.

Assessing the impact of processing therefore requires analytical approaches with greater structural specificity than the spectrophotometric assays. Total phenolic content, total flavonoid content, and common antioxidant assays are useful screening tools, but they cannot determine whether a post-treatment signal reflects the same native compound, a more extractable constitutive compound, or a newly formed oxidation or nitration product [1,2]. Non-MS chromatographic readouts improve separation but are often insufficient for definitive isomer discrimination or product confirmation. LC–MS/MS and high-resolution mass spectrometry are therefore needed because they combine chromatographic separation with mass-resolved and fragmentation evidence to track compound identity, product formation, and technology-specific marker changes [2]. A similar principle applies when chemical change is inferred from sensory shifts. Distinguishing thermolabile essential-oil constituents from their degradation products depends on structurally resolved analysis rather than non-specific aggregate indices [8]. Yet even when LC–MS is employed, comparability across studies is often constrained by differences in extraction conditions, chromatographic coverage, ionization and acquisition strategies, annotation confidence, and reporting completeness. Such methodological variation can shift an observation from being interpreted as a transformation to release, or vice versa, without any genuine inconsistency in the underlying chemistry. Thus, the field faces an evidence-driven gap: the literature on non-thermal processing and phenolics is extensive, but the basis for distinguishing what is preserved, what becomes more extractable, what undergoes oxidation, and what experiences structural conversion remains uneven.

This review addresses this gap by synthesizing recent studies through an LC–MS-centered evidential framework. The aim is to align data interpretation with compound identification confidence and enable more robust cross-technology comparisons. Specifically, it integrates compound-resolved evidence to elucidate how major non-thermal processing technologies reshape the polyphenol profile across food and beverage matrices. It also critically examines how profiling and fingerprinting strategies support or limit claims of molecular modification. By adopting this evidential perspective, this review emphasizes the distinction between apparent stability arising from changes in extractability and genuine chemical evolution associated with release, oxidation, and structural conversion processes.

To ensure that synthesis remains aligned with evidential strength, this review is limited to food and beverage matrices processed using non-thermal technologies and does not consider pharmacological applications based on isolated pure compounds. The literature was identified through Scopus and Web of Science (search completed 5 March 2026) using six technology- and topic-specific search strings combining the following terms: “ultrasound,” “ultrasonic,” “high pressure processing,” “HPP,” “high hydrostatic pressure,” “pulsed electric field,” “PEF,” “cold plasma,” “non-thermal plasma,” “plasma-activated water,” “phenolic,” “polyphenol,” “flavonoid,” “LC–MS,” “LC–MS/MS,” “UPLC–MS,” “metabolomic,” “profiling,” “digestion,” “bioaccessibility,” “bioavailability,” “gut microbiota,” “process optimization,” “quality assurance,” and “food.” All searches were restricted to English-language original research articles published between 2021 and early 2026. A total of 958 records were retrieved from Scopus across the six search strings; additional records were identified through Web of Science using equivalent queries and through citation tracking of authoritative food-chemistry reviews. After removal of duplicates and records outside the scope of this review, 382 records were screened by title and abstract. Of these, 196 records were assessed by full-text review. Studies employing LC–MS or LC–MS/MS for compound-specific phenolic characterization in processed food or beverage matrices were included; pharmacological studies using isolated pure compounds, studies reporting only total phenolic or antioxidant indices without chromatographic resolution, and non-food applications were excluded. A final set of 111 studies was retained and forms the evidence base of this review. Greater interpretive weight was given to studies employing LC–MS/MS or high-resolution mass spectrometry for compound-resolved evidence, whereas total phenolic content, total flavonoid content, and antioxidant assays were treated only as supportive context.

The manuscript is organized to follow the inferential logic established above. It first clarifies what constitutes LC–MS profiling and fingerprinting. It then explains how identity confidence, quantitative reliability, and reporting sufficiency shape the conclusions that can be drawn from observed compositional shifts. The review also summarizes technology-specific evidence across four non-thermal categories—ultrasound, high-pressure processing, pulsed electric fields, and non-thermal plasma. These four technologies represent the most extensively studied non-thermal methods in polyphenols’ research. The approach of using and reviewing the LC–MS-based evidence for the comparison of polyphenols structure and function has not been adopted previously [12,13]. The selection reflects evidence of availability rather than industrial prevalence alone. The findings are then integrated into a cross-cutting transformation map that distinguishes release-driven changes from oxidation and structural conversion processes. Finally, the review extends from molecular fingerprints to digestion-related fate and functional outcomes and outlines how LC–MS evidence can support process optimization and industrial quality assurance while retaining explicit evidential limits.

## 2. LC–MS Evidence Framework

### 2.1. Defining LC–MS Polyphenol Characterization: Targeted, Widely Targeted, and Untargeted Workflows

LC–MS-based polyphenol characterization uses overlapping terms that describe either workflow design or identification confidence, and the two should not be conflated. The terms targeted, widely targeted, and untargeted describe how analytes are surveyed. Confirmed identity, putative annotation, and tentative identification (corresponding to Tier A, B, and C as defined in Section 2.2, respectively) describe how securely a feature is assigned. The central methodological question is therefore not semantic but inferential: when do LC–MS data support claims of preservation, release, or true structural conversion?

Targeted workflows generally quantify a predefined set of analytes using authentic reference standards and calibration-based approaches. Results are reported as absolute concentrations. When appropriate validation is documented, such designs allow relatively robust statements regarding both the direction and magnitude of change in the measured compounds. For example, targeted analyses of apple juice commonly include compounds such as chlorogenic acid. In contrast, targeted quantification in sesame oil typically focuses on a defined panel of trace phenolics. These studies are usually accompanied by reported performance characteristics, including sensitivity, linearity, and related analytical metrics [14,15]. Targeted strategies are likewise employed when specific analytes constitute the primary analytical objective. One example is the pharmacokinetic measurement of vanillic acid in plasma. Another is the quantification of individual constituents, such as icariside and psoralen, in complex food matrices [16,17]. Nutrient-oriented assays may similarly adopt targeted LC–MS/MS for selected compounds (e.g., quercetin, curcumin). In such cases, solid-phase extraction is often included in sample preparation [18]. The limitation most relevant to transformation studies is structural. Because the analyte list is fixed, targeted methods can reliably track changes in the compounds being monitored. At the same time, they are not designed to detect unexpected derivatives or secondary products. Under these conditions, statements such as “no new products detected” are more likely to indicate the limits of the analytical approach than the absence of chemical change.

By contrast, untargeted profiling and fingerprinting are typically used as broad surveys that rely on multivariate patterns for classification, marker screening, or comparative assessment [19,20]. In this article, the term “polyphenol profiling” is used to describe the reporting of large sets of detected features and their associated annotations. Such analyses primarily aim to document compositional coverage rather than to establish discriminative structure. In comparison, “metabolomic fingerprinting” refers to approaches that focus on multivariate patterns for sample discrimination. These patterns are typically used for grouping, classification, or authentication purposes [21,22]. These approaches paired with chemometrics can separate foods and botanicals by geography, processing history, or formulation [20,22]. They also appear in functional or predictive contexts, including marker-based prediction of antioxidant potential in honey and identification of discriminant non-volatile metabolites associated with tea processing techniques [23,24], as well as screening phenolic antioxidants in *Persicaria chinensis* alongside bioactivity assessment [25]. The inferential boundary is clear: fingerprinting provides strong evidence that samples differ, but on its own, it does not demonstrate that specific polyphenols have undergone structural transformation unless the discriminant features are chemically resolved. This distinction matters for synthesis, as multivariate shifts may also arise from extraction selectivity, ionization effects, or variability in annotation.

Between narrow targeting and fully untargeted workflows, “widely targeted” strategies expand coverage while retaining predefined analyte panels, as illustrated by comparisons of polyphenolic patterns during *Rosa xanthina* fruit development [26]. These hybrid designs can enhance analytical coverage without sacrificing standardization, thereby strengthening within-study comparability. Nevertheless, they remain constrained by the predefined monitoring list and cannot fully capture transformation products that fall outside this panel.

### 2.2. Evidential Criteria for Transformation Inference: Identification Confidence, Quantitative Reliability, and Inferential Scope

Transformation claims are most defensible when LC–MS evidence supports comparisons at the compound level rather than relying solely on profile-level separation [27,28]. Because transformation is mechanistic language, it implies that specific structures are altered, degraded, conjugated, or formed. For this review, identification support is grouped into three tiers. Tier A denotes confirmed identity against an authentic standard, supported by accurate mass, retention time, and available MS/MS agreement. Tier B denotes MS/MS-supported putative annotation without standard confirmation. Tier C denotes database-level or tentative feature assignment lacking confirmatory MS/MS evidence. Mechanistic language is scaled to the highest identification support available. This tiered framework provides a consistent basis for scaling mechanistic claims to the level of analytical evidence available, rather than serving as an absolute quality certification for individual studies.

At the highest level of evidentiary support, some studies confirm identity by matching retention time and MS/MS spectra to authentic standards for specific analytes, including catechin and hydroxytyrosol in validated measurements [27]. Such confirmation offers the clearest basis for interpreting increases, decreases, appearance or disappearance as changes in defined compounds rather than shifts in ambiguous features. Standards are also described in pharmacokinetic investigations reporting identification of compound classes such as triterpenoid saponins and flavonoids in *Astragalus membranaceus* plasma samples [29]. However, compound-class reporting leads to uneven transformation inference unless specific entities, including isomers, are distinguished.

When reference standards are unavailable, studies often rely on accurate mass measurements. Diagnostic fragmentation is used and supported by spectral libraries or defined interpretive strategies. Examples include feature-based molecular networking applied to proanthocyanidins and fragmentation-based approaches used to distinguish glycosylated flavones in tea matrices [30,31]. These approaches can strengthen plausibility, particularly when decision rules are transparent. Yet structural ambiguity often remains: isomer discrimination can be challenging under certain annotation conditions, even with high-resolution data [32,33]. This is consequential for interpretation. Apparent emergence or depletion of a “compound” may reflect unresolved isomer mixtures, co-elution, or shifting ionization rather than genuine structural conversion. In other metabolomic workflows, features are assigned primarily via database matching with limited fragmentation support, and such studies commonly acknowledge reduced structural certainty [28,34]. As a result, papers can differ substantially in evidential strength even when they adopt similar transformation terminology.

A second criterion relates to quantitative reliability and comparability when differences in abundance are used to support transformation claims [14,35]. Targeted quantification studies typically report concentration data together with validation parameters. These include linearity, limits of detection and quantification, and recovery, as shown in studies of phenolics in apple juice [14]. Quantitative workflows applied to complex matrices, such as rat plasma, also commonly report the use of internal standards [35]. These methodological details are important. Matrix effects and batch-to-batch variability can substantially influence signal intensity. In highly complex biological matrices, authors frequently note sources of variability and analytical challenges that complicate interpretation of LC–MS data. This point is illustrated by studies of urine metabolites derived from functional foods [36]. Quantitative reliability in such studies is often limited or not fully documented. Under these conditions, observed changes in abundance should be interpreted as tentative signals. They should not be taken as direct evidence of chemical conversion.

A third criterion concerns how LC–MS changes are linked to “transformation” versus functional association [37,38]. Several studies interpret profile changes alongside functional readouts, including digestion-related bioaccessibility or bioactivity measures [37]. Multi-platform profiling has also been used with functional endpoints such as anticholinesterase activity, although the extent to which specific transformation products are independently established varies across designs and reporting practices [38]. For synthesis, two forms of support should be kept distinct: compound-resolved evidence that can substantiate transformation mechanisms, and profile-level correlations that are useful for hypothesis generation but do not establish structural change by themselves [28,34]. This distinction informs how different types of evidence are treated in the sections that follow.

### 2.3. Sources of Inter-Study Heterogeneity and Limits to Comparability

Cross-study synthesis is constrained by heterogeneity in extraction, detection, and annotation, which shapes what changes are even observable [36,39]. This heterogeneity does more than add noise and it can create apparent contradictions when differences in analytical visibility are interpreted as differences in chemistry.

Extraction strategy is a major driver because recovery depends on matrix composition and solvent selectivity, as illustrated in work on solid residues from the essential oil industry [39]. Under such conditions, a reported “loss” may be indistinguishable from reduced extractability unless recovery is evaluated. Processing history adds another layer: fermentation-associated changes reported for Fuzhuan brick tea plausibly reflect biochemical remodeling, while the magnitude and direction of reported shifts are difficult to compare across studies without aligned baselines and analytical pipelines [40]. In other words, processing may be causal but is still being confounded by methodological non-equivalence.

Platform differences further limit comparability. High-performance thin-layer chromatography (HPTLC), for instance, has different separation and detection characteristics than UHPLC–MS-centered workflows, constraining cross-platform comparison of profiles [41]. Even within LC–MS studies, key parameters are not always reported consistently. Some papers specify ionization modes and discuss matrix-related considerations, yet others provide too little detail to judge whether differences reflect chemistry or instrumentation [15,35,36]. From a synthesis perspective, the implication is pragmatic. Claims about transformation trajectories across foods and processing conditions are conditional on methodological comparability. Where comparability cannot be established, the safer conclusion is “processing-associated variation” rather than convergent evidence for specific transformation pathways.

### 2.4. Implications for Evidence-Weighted Synthesis and Reporting Sufficiency

**Figure 1:**
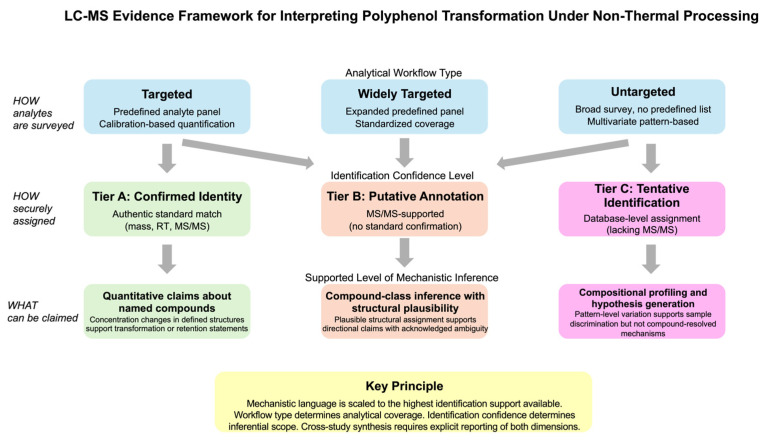
LC–MS evidence framework for interpreting polyphenol transformation under non-thermal processing. The framework separates analytical workflow type (targeted, widely targeted, untargeted) from identification confidence (Tier A: standard-confirmed identity; Tier B: MS/MS-supported putative annotation; Tier C: database-level tentative assignment). Each combination is linked to a defined level of mechanistic inference: Tier A supports quantitative claims about named compounds; Tier B supports compound-class inference with structural plausibility; Tier C supports compositional profiling and hypothesis generation only.

Collectively, Section 2.1, Section 2.2 and Section 2.3 show that the strength of transformation inference is bounded not only by workflow intent but also by what can be independently evaluated from the report itself. Confidence of identification cannot be judged without adequate chromatographic and MS/MS context, particularly where isomeric complexity and feature ambiguity are plausible [14,22,28,31]. Abundance-based arguments are similarly fragile when matrix effects and validation practices are unclear, especially in complex food or biological matrices where recovery and ion suppression can shape apparent trends [14,35,36]. Cross-study inconsistencies also become interpretable only when extraction and acquisition parameters are reported in sufficient detail to separate methodological visibility from underlying chemistry [18,39]. In synthesizing this literature, studies are therefore not treated as equally informative: where reporting prevents appraisal of identity or comparability, conclusions are scaled to profile-level variation and hypothesis generation, and not to compound-resolved transformation [19,30]. In Section 3, this logic is applied to differentiate compositional changes arising from enhanced release or extractability from those associated with oxidative or nitrative modification. Claims of structural conversion are reserved for cases supported by the detection of identifiable products or adducts, with interpretive weight scaled to the level of identification certainty. The overall logic of this evidence framework, linking workflow type, identification confidence, and supported level of mechanistic inference, is summarized in Figure 1.

## 3. Technology-Specific Alterations of Phenolic Profiles

*Scope*

This section examines how four non-thermal processing categories—ultrasound, high-pressure processing (HPP), pulsed electric fields (PEF), and non-thermal plasma (NTP)—shape the stability and transformation of phenolic compounds. These technologies were selected because they are widely studied, increasingly used in food processing, and, within the present review scope, provide the most interpretable LC–MS(/MS)-based evidence for comparing release/extractability, retention, oxidation risk, and structural conversion across food and beverage matrices [12,13]. The selection reflects evidence availability rather than industrial prevalence: other non-thermal approaches such as UV-C, pulsed light, ozone, and supercritical CO_2_ can also modulate phenolic profiles but are positioned as priorities for future LC–MS-enabled investigation, as discussed below. This evidence base enables structure-level interpretation and reduces reliance on shifts inferred solely from global indices.

Other non-thermal or mild approaches, such as ultraviolet C (UV-C) or ultraviolet (UV) treatment, pulsed light, ozone and plasma-activated water, ionizing irradiation, and supercritical CO_2_, can also modulate phenolic profiles and warrant dedicated synthesis. However, for many of these technologies, phenolic outcomes are more frequently reported using total phenolic or flavonoid indices, bulk antioxidant assays, or narrow targeted panels. MS/MS confirmation is not consistently provided, and quantitative comparability across studies is often limited. These constraints hinder integration into the evidence-weighted LC–MS framework applied here. Accordingly, these approaches are positioned as priorities for future LC–MS-enabled investigation and reporting standardization, rather than as core evidence streams in the present technology-by-technology synthesis.

In line with the evidential criteria outlined in Section 2, this synthesis prioritizes studies employing LC–MS or LC–MS/MS platforms that enable compound-specific tracking. Studies based solely on total phenolic content or total flavonoid content assays are used only to contextualize broad extractability trends because such colorimetric methods cannot distinguish structural preservation from degradation products or non-phenolic interferences [42]. The evidence base is also uneven: HPP and ultrasound studies are dominated by extraction and preservation studies in complex matrices, whereas the NTP literature more frequently provides direct evidence for abiotic chemical transformations such as oxidation and nitration in model systems and food matrices [43,44]. To orient the technology-by-technology synthesis, Table 1 summarizes the representative LC–MS studies included in this review, together with evidence-tier assignments linked to the framework defined in Section 2.2.

Tier assignments reflect the predominant identification approach reported in each cited study, mapped to the framework defined in Section 2.2. Assignment at the individual compound-feature level may differ and should be evaluated against the original articles when compound-specific mechanistic conclusions are drawn.

Taken together, the evidence inventory shows that the literature is uneven across technologies. HPP and ultrasound studies are dominated by retention and release/extractability patterns in complex food matrices. PEF occupies an intermediate position because release may coexist with oxidation risk, and NTP provides the clearest examples of direct chemical conversion. Cross-technology comparison should therefore be weighted by analytical confidence rather than by study count alone. These patterns are examined in further detail in the technology-specific sections below and integrated in the cross-cutting synthesis in Section 3.5.

### 3.1. Ultrasound Processing

#### 3.1.1. Typical Process Windows

Ultrasound appears predominantly as an extraction-aid technology. Operating frequencies were typically fixed (e.g., 20 kHz or 37 kHz), while power inputs varied widely (approximately 100 W to 4000 W) depending on scale. Treatment times ranged from short pulses of 2–10 min [42] to extended extraction periods of 30–60 min [64]. Temperature control was routinely emphasized. Several studies used water baths or cooling systems to maintain 25 °C to 50 °C, thereby separating acoustic cavitation effects from secondary thermal contributions [65,66]. However, specific dissipation rates of ultrasonic energy into heat were not consistently reported.

#### 3.1.2. Fingerprint Trends

A dominant trend observed across ultrasound-treated matrices, including giloy leaves [51], blackthorn fruit [52], and strawberry tree fruit [53], is a statistically significant increase in the detected abundance of intracellular phenolics. For example, optimized ultrasound treatment (22.5:1 solvent ratio, 40 min) maximized the yield of flavonoids and phenolic acids [51]. In *Olea europaea* leaves, ultrasound extraction yielded higher concentrations of oleuropein and luteolin-7-O-glucoside than shaking water baths [65]. At higher sonication amplitudes (>60%) or with extended treatment times, anthocyanin content in *Erythrina crista-galli* flowers reached a plateau or declined [66], a pattern consistent with observations in other ultrasound-assisted extraction studies where excessive energy input reduced polyphenol recovery [64,67]. This pattern suggests that sonication intensity and duration can shift from release-promoting to degradation-promoting in polyphenol-rich matrices, though the specific threshold is matrix-dependent and requires empirical determination for each system.

#### 3.1.3. Confirmed vs. Plausible Transformations

The available evidence supports enhanced release or extractability rather than de novo synthesis in the reviewed ultrasound studies [48]. Acoustic cavitation disrupts cell walls, enhances mass transfer, and promotes the release of bound phenolics. In wheat bran, ultrasound extraction liberated ferulic acid and other bound phenolics, while LC–MS profiles showed no evidence for the formation of novel derivatives not present in the untreated material [54]. Although high-intensity sonolysis can, in principle, generate reactive species such as hydroxyl radicals and promote degradation, the optimization-focused studies reviewed here generally operated under conditions designed to minimize such effects [67].

#### 3.1.4. Matrix Dependence

Matrix resistance determines ultrasound efficacy. Hard tissues such as bark required higher energy inputs or combined solvents to achieve release comparable to that of soft tissues such as berries [68]. In liquid systems such as rose-distillation side streams, ultrasound effectively recovered residual phenolics without significantly altering the overall profile [69]. Overall, the reviewed ultrasound literature is dominated by release/extractability signals under the controlled low-temperature conditions most employed. This pattern reflects the optimization focus of the field rather than a comprehensive mechanistic survey: systematic studies tracking specific degradation products or novel adducts under higher-intensity or longer-duration sonication remain sparse. A key open question for future work is whether degradation onset can be predicted from process parameters alone, or whether matrix composition, particularly cell-wall architecture and endogenous enzyme activity, must also be modeled to define safe operating windows for each food system.

### 3.2. High Hydrostatic Pressure (HPP)

#### 3.2.1. Typical Process Windows

HPP treatments were characterized by pressures between 300 MPa and 600 MPa and holding times ranged from 3 to 15 min [45,49]. Starting temperatures were generally controlled at ambient or refrigerated levels (4–25 °C), with some studies monitoring adiabatic heating [47]. Some articles compared HPP directly to thermal treatments (UHT, HTST) to isolate pressure-specific effects [50,70].

#### 3.2.2. Fingerprint Trends

HPP generally maintained phenolic profiles highly similar to fresh controls. In blueberry juice, widely targeted metabolomics (WTMs) showed that while thermal processing significantly upregulated degradation products (e.g., 1,3,5-benzenetriol), HPP samples clustered closely with fresh juice [45]. Similarly, it was found that HPP (400–600 MPa) preserved the anthocyanin and flavonol profile of spine grape juice, whereas mild heating induced detectable changes [46]. However, HPP (600 MPa/10 min) significantly increased the detectable levels of naringenin chalcone and other flavonoids in tomato juice, attributing this to enhanced extractability from the pulp [48]. The consistent clustering of HPP samples with fresh controls across these studies is analytically significant: it demonstrates not only preservation of constitutive compounds but also the absence of the oxidation and condensation products that characterize thermally processed matrices. However, this pattern should be interpreted with the caveat that most reviewed studies used untargeted or widely targeted platforms at Tier B confidence, which may miss low-abundance transformation products if they fall below detection thresholds.

#### 3.2.3. Confirmed vs. Plausible Transformations

The evidence overwhelmingly supports release/extractability and retention over transformation. Detailed profiling of raspberry juice [70] and blended juices [47] indicates that HPP does not induce the extensive hydrolysis or polymerization observed during thermal processing. A notable exception involves phloretin xyloglucoside in cloudy apple juice, where multi-pulsed HPP showed different retention patterns from static HPP [49]. The precise mechanism is not established from this study alone; pressure-cycle-dependent effects on phenolic oxidation or matrix release kinetics are plausible explanations, and a mechanistic account would require dedicated enzyme-kinetics data beyond the scope of this review.

#### 3.2.4. Matrix Dependence

The physical state of the matrix (clear juice versus cloudy juice or puree) strongly influences the HPP effect. In cloudy juices or smoothies containing particulate matter [48,71], HPP caused a leaching effect and increased the soluble fraction of phenolics by disrupting residual cell structures. In clear juices, this effect was negligible [45]. Taken together, the HPP literature most consistently supports structural retention plus release from particulate matrices. The key open question is whether pressure-cycle design (multi-pulsed versus static) can be tuned to control the release/oxidation balance in cloudy matrices containing active oxidoreductases, a question that currently lacks sufficient compound-resolved evidence across multiple food systems.

### 3.3. Pulsed Electric Fields (PEFs)

#### 3.3.1. Typical Process Windows

PEF applications utilized field strengths ranging from 0.5 kV/cm to 35 kV/cm, with specific energy inputs typically between 1 kJ/kg and 20 kJ/kg [55,72]. Treatments were applied to pumpable fluids (juices) or wet solids (pretreatment for drying/extraction). Pulse width and frequency varied and reported operating conditions generally avoided the bulk heating characteristic of conventional thermal processing, although exact limiting temperatures were not always reported consistently across studies [73].

#### 3.3.2. Fingerprint Trends

Metabolomic data for PEF are highly matrix-dependent, and this dependence follows a pattern that can be understood mechanistically. In liquid or semi-liquid matrices where electroporation directly disrupts cell membranes without subsequent high-temperature exposure, PEF typically increases phenolic extractability. In pineapple-flesh extraction, PEF combined with ultrasound increased the recovery of polyphenols and their derivatives [56], and in apple-pomace extracts, PEF treatment (3 kV/cm, 5 kJ/kg) preserved phenolic profiles at a level comparable to untreated samples and superior to thermal treatment [73]. In contrast, when PEF is used as a pretreatment before a drying step, the balance shifts. During prune drying, PEF pretreatment led to significant decreases in total phenolics, flavonoids, and antioxidant capacity, with specific degradation of anthocyanins [55]. This sequence-dependent outcome illustrates why PEF effects cannot be evaluated from the PEF step alone: the downstream process is a co-determinant of the final phenolic profile.

#### 3.3.3. Confirmed vs. Plausible Transformations

The primary mechanism is release via electroporation. Using ultra-performance liquid chromatography coupled with ion mobility spectrometry–mass spectrometry, PEF enhanced the release of flavanones from orange peel without altering their isomeric forms [58]. However, true transformation via oxidation remains a documented risk. Widely targeted metabolomic evidence indicates that intense PEF treatment of prunes downregulated anthocyanins, plausibly because electrochemical reactions and/or increased exposure to endogenous oxidases followed membrane rupture. The oxidases implicated include polyphenol oxidase and peroxidase, although specific oxidative derivatives were not structurally isolated in the cited study [55].

#### 3.3.4. Matrix Dependence

The conductivity and physical structure of the matrix are decisive. In liquid orange-juice processing [58], PEF facilitated efficient extraction. In solid matrices such as broccoli by-products [56] or prunes [55], the outcome depended strongly on the downstream process step. PEF enhanced the release of phenolic metabolites in broccoli by-products [57] but accelerated oxidative loss during hot-air drying of prunes [55]. Therefore, PEF remains release-dominant, but downstream oxidation risk is more matrix- and process-dependent than in HPP or ultrasound.

The defining open question for PEF is how to predict whether electroporation will promote extractability or trigger oxidative cascades, given that this outcome depends on the combination of field strength, matrix conductivity, and what enzymatic or chemical processes follow membrane disruption.

### 3.4. Non-Thermal Plasma (NTP)

#### 3.4.1. Typical Process Windows

NTP treatments included dielectric barrier discharge, plasma jets, and plasma-activated water. Voltages ranged from 6 kV to 80 kV, with treatment times from seconds to 30 min [44,62]. Operating gases included air, argon, helium, and nitrogen. These systems are generally described as non-thermal because they avoid the bulk heating characteristic of conventional thermal processing; however, no single fixed operating temperature applies across dielectric barrier discharge, plasma jet, and plasma-activated water studies, and temperature reporting was not uniform across the cited literature [43].

#### 3.4.2. Fingerprint Trends

NTP generated the most complex metabolomic shifts. A distinct reduction in flavan-3-ols and an increase in dihydrochalcones and flavonols in plasma-treated apple slices were reported [44]. In model systems, the degradation of phenolic acids (e.g., chlorogenic acid) and the formation of new peaks were observed. But in fresh-cut strawberries, mild plasma treatment induced the accumulation of phenolics and activated the phenylpropanoid pathway [60].

#### 3.4.3. Confirmed vs. Plausible Transformations

NTP is unique in driving true abiotic chemical transformations. Within the evidence summarized here, LC–MS support is strongest for the appearance of new features with mass shifts consistent with oxidation and nitration relative to the parent phenolics [43,61]. In model systems, the formation of new LC–MS peaks following plasma treatment of phenolic acids and the mass differences observed are consistent with nitro-group addition (net +45 Da), although compound-by-compound MS/MS fragment assignment for specific nitrated isomers was not reported [43]. In apple slices, the selective loss of flavan-3-ols was attributed to plasma-induced oxidative stress [44], supported by the disappearance of specific catechin and epicatechin signals in the LC–MS profile. In living tissues such as fresh-cut produce, observed phenolic increases are more likely to reflect physiological stress responses than direct abiotic chemical synthesis [60,74], a distinction that requires careful experimental design to resolve. The available evidence therefore supports classifying NTP-induced changes as direct chemical transformations in non-living or model matrices, and as a mixture of abiotic and biotic responses in metabolically active tissues.

#### 3.4.4. Matrix Dependence

Surface area and water content are key determinants. NTP effects are surface-limited, with penetration depths in the micron range. In dry powders, NTP reduced microbial load with minimal changes to curcuminoids compared with irradiation [75]. In aqueous systems such as plasma-activated water, acidification and the accumulation of H_2_O_2_ and nitrate drive reactions that differ from gas-phase treatments [63,76]. Compared with other non-thermal technologies, NTP shows the clearest technology-specific evidence for direct chemical conversion, but outcomes remain highly dependent on gas chemistry, exposure mode, and whether the matrix remains metabolically active. The key open question is establishing the exposure conditions under which nitration shifts from trace-level to food-safety-relevant, a distinction that requires quantitative compound-resolved data currently absent from most reviewed studies.

### 3.5. Cross-Technology Snapshot

**Figure 2:**
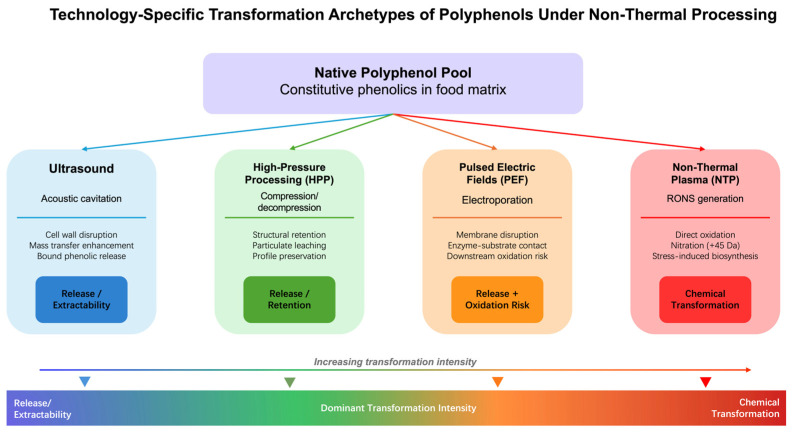
Technology-specific transformation archetypes of polyphenols under non-thermal processing, based on LC–MS evidence reviewed in Section 3.1, Section 3.2, Section 3.3 and Section 3.4. Ultrasound and high-pressure processing (HPP) are predominantly associated with release and enhanced extractability of constitutive phenolics through mechanical cell disruption. Pulsed electric fields (PEFs) follow the same release logic via electroporation but show greater matrix- and process-dependent oxidation risk, particularly when membrane disruption is followed by oxidase-active downstream steps. Non-thermal plasma (NTP) stands apart by generating reactive oxygen and nitrogen species, producing the clearest LC–MS evidence for direct oxidation, nitration, and stress-induced biosynthetic responses among the four technologies.

The reviewed technologies can be grouped by dominant effect, but this classification is evidential rather than absolute. HPP and ultrasound are grouped as release/extractability-dominant because the cited studies mainly report preservation of constitutive compounds together with higher detectable abundance. PEF occupies an intermediate position because it often follows the same release logic but shows stronger matrix- and process-dependent oxidation risk. NTP stands apart because the reviewed studies more frequently report direct oxidation or nitration products and stress-related responses. This divergence shows that non-thermal is an insufficient descriptor for predicting chemical fate, and that a more granular transformation map is required. These technology-specific transformation archetypes and their position along the release-to-conversion gradient are illustrated in Figure 2.

## 4. A Cross-Cutting Transformation Map

*Integrative Scope*

Building on the technology-specific evidence in Section 3, this section integrates the findings into a generalized map of phenolic transformations under non-thermal processing conditions. By prioritizing high-resolution LC–MS evidence over generic colorimetric assays, a distinction is drawn between apparent changes driven by extractability and true molecular transformations supported by compound-resolved evidence. The analysis indicates that although thermal energy is replaced by mechanical, electrical, or chemical inputs, the resulting metabolomic alterations converge on a limited number of archetypal patterns: compound release, oxidation, and specific structural transformations.

### 4.1. Stability vs. Apparent Stability

A recurring theme across HPP and ultrasound studies is the increase in post-processing phenolic signals. In the cited studies, these higher signals were reported for compounds already present in untreated materials; the available evidence therefore supports enhanced release or extractability rather than de novo synthesis in the reviewed HPP and ultrasound studies. For example, HPP-treated tomato juice showed higher flavonoid titers than fresh controls, while untargeted profiling indicated that the molecular species remained constitutive [48]. By contrast, apparent stability in total phenolic assays can mask underlying structural changes. A PEF study reported maintenance of bulk antioxidant values even though individual polyphenols fluctuated, underscoring the need for chromatographic resolution [73].

### 4.2. Release and Repartitioning

The release archetype is the dominant mechanism across ultrasound, HPP, and PEF. Mechanical disruption (cavitation in ultrasound; compression/decompression in HPP) or electrical disruption (electroporation in PEF) compromises cellular compartments and cell walls, shifting phenolics from the solid phase into the solvent or serum fraction. Accordingly, ultrasound treatment increases the partitioning of phenolics toward the extraction medium [51,65], and HPP similarly increases the soluble fraction of pulp-associated phenolics in blended juices [47]. This repartitioning can enhance bioaccessibility [71], but it may also expose previously compartmentalized phenolics to endogenous oxidases, creating conditions for degradation when enzymes are not simultaneously inactivated [74].

### 4.3. Oxidation Across Technologies

Oxidation represents a major pathway for true transformation. In NTP-treated systems, oxidation constitutes the most direct threat because of the presence of exogenous reactive oxygen and nitrogen species (RONS). One study demonstrated the selective depletion of flavan-3-ols in apples, a class of compounds that is highly susceptible to oxidative reactions [44], while another work confirmed that plasma-processed air can oxidize model phenolic acids [43]. In contrast, oxidation in PEF and HPP systems is typically indirect. By rupturing cellular membranes, these technologies facilitate enzyme–substrate contact and thereby promote enzyme-mediated oxidation. For example, anthocyanin degradation was observed in PEF-pretreated prunes, which was attributed to enhanced enzymatic activity during the subsequent drying phase [55]. It should be noted that some reports of “oxidation” may reflect measurement artifacts if LC–MS/MS conditions, such as source temperature, are not strictly controlled, although the cited studies generally implemented appropriate analytical controls.

### 4.4. Structural Conversions

Beyond simple oxidation, specific structural conversions are rare but have been documented. Nitration appears to be unique to NTP and represents a crucial safety consideration for its applications, as mass spectral evidence has demonstrated the nitration of phenolic rings in model systems exposed to plasma-generated nitrogen species [43]. Deglycosylation is well established in thermal processing, where glycosides are readily hydrolyzed, whereas HPP and PEF generally preserve glycosidic bonds, with anthocyanin glycosides reported to remain intact under high pressure, thereby differentiating these technologies from thermal sterilization [45,46,70]. Polymerization has not been explicitly characterized by molecular weight in all studies, but the observed loss of monomeric flavanols in NTP-treated systems and the changes in procyanidin fractions in apple juice suggest possible oligomerization or binding to cell wall components [44,49].

### 4.5. Matrix Dependence as a First-Order Driver

The physical state of the food matrix governs the transformation pathway. In liquid juice systems, HPP tends to act homogeneously and largely preserves phenolic profiles, whereas in solid or particulate matrices, including smoothies and purees, it functions more as an extraction force that enhances the release of matrix-bound phenolics [42,45]. The living status of the matrix further modulates these pathways. In metabolically active tissues, treatments including NTP or mild ultrasound can trigger physiological biosynthetic responses, whereas in processed or non-living matrices, including powders and juices, only abiotic reactions such as degradation or release are observed [60,75]. In addition, anthocyanin stability is strongly pH-dependent, and technologies that modify pH, such as plasma-activated water, can indirectly alter phenolic speciation and color stability [63].

### 4.6. Toward Process Marker Panels

To standardize the assessment of non-thermal effects, a shift from total phenolic content toward specific marker panels is required. Based on the available evidence, robust panels should incorporate sensitivity markers, defined as compounds that degrade rapidly and therefore reflect process severity, including cyanidin-3-glucoside as an indicator of thermal or oxidative stress [55]. Release markers should also be included to capture cell-disruption effects, particularly cell wall-bound or otherwise inaccessible compounds that increase only after matrix breakdown, such as ferulic acid and quercetin aglycones [54]. Transformation markers are necessary to identify technology-specific chemistry. Within the reviewed NTP literature, nitrated phenolic derivatives are the most technology-specific candidate markers because they arise from plasma-associated reactive nitrogen species [43]. By contrast, Maillard-related intermediates function as thermal counter-markers; their absence in HPP-treated blueberry juice, compared with thermally processed juice, supports successful avoidance of heat-driven reactions [45]. Finally, all proposed markers require validation with MS/MS fragmentation evidence rather than retention time alone to minimize isomer misassignment [58].

### 4.7. Robust vs. Plausible Synthesis

Robust evidence indicates that HPP, ultrasound, and PEF can enhance the detectable abundance of constitutive phenolics while largely avoiding the extensive thermal degradation associated with conventional heating [45,51,56]. NTP more often produces direct oxidation- or nitration-related changes, although the extent of conversion remains strongly matrix-dependent [44]. Accordingly, claims that non-thermal technologies increase phenolic content should be interpreted as reflecting enhanced extractability or bioaccessibility unless identifiable products or adducts support true structural conversion.

## 5. Linking LC–MS Molecular Fingerprints to Biological Relevance

The relevance of the transformation map extends beyond composition because processing-induced changes in molecular form, extractability, and matrix binding determine what survives digestion, what becomes bioaccessible, and what enters microbial metabolism. LC–MS-based untargeted metabolomics can annotate hundreds of compounds in processed food matrices, but the biological relevance of these fingerprints ultimately depends on what happens during gastrointestinal digestion. A consistent gap emerges between the high diversity of phenolic features detected in food extracts and the narrower subset of metabolites that persist in forms most likely to contribute to physiological effects.

### 5.1. Gastrointestinal Digestion: Stability, Release, and Transformation

Evidence to date indicates that phenolic fingerprints do not behave uniformly during GI transit. Their stability is highly selective and strongly dependent on the surrounding matrix. A common pattern is a marked reduction in measurable native polyphenols when conditions shift from the gastric to the intestinal phase, attributed to pH-driven instability and interactions with digestive enzymes and other luminal components.

For a broader trend, subclass-specific behavior is apparent. In honey, phenolic acids such as caffeic acid and *p*-coumaric acid appear comparatively stable during the gastric phase. But they can drop substantially during the intestinal phase, with losses reported up to 44%, which is consistent with alkaline pH sensitivity typical of hydroxycinnamic acids [77]. A similar selectivity is seen in tiger nut beverages: resveratrol and chlorogenic acid decrease significantly after intestinal digestion, while metabolites such as homovanillic acid emerge, suggesting degradation or transformation of precursor structures under digestive conditions [78]. Anthocyanins, which are generally unstable at neutral pH, illustrate how matrix context can alter apparent recovery, as polysaccharide gums (xanthan and guar gum) added to *Aronia melanocarpa* purées significantly affected anthocyanin recovery after digestion (Tier A/B), plausibly by restricting diffusion of digestive fluids or by chelating ions that catalyze degradation [79].

Another recurring theme is the importance of the “bound” phenolic fraction, which is often underrepresented by solvent-based extractions but becomes relevant as digestion progresses. Metabolomics (Tier B) indicated that free phenolics in wheat flour are rapidly released or degraded, while bound ferulic acid derivatives in wheat bran resist gastric conditions and require intestinal enzymatic hydrolysis or colonic fermentation for release [80]. A comparable pattern was reported in oat bran, where most bound polyphenols were not released during the gastric phase but became progressively solubilized during the intestinal and colonic stages [81].

Processing- and matrix-induced covalent modifications add a further layer of complexity because they can redirect “digestion fate” toward conjugated forms that are rarely captured in standard bioaccessibility workflows. Tier A/B evidence showed that 4-methylcatechol can covalently bind to β-lactoglobulin via thiol–quinone addition and that proteolytic digestion releases specific cysteine–phenolic adducts, supporting the interpretation that apparent “losses” of polyphenols in protein-rich matrices may reflect sequestration and re-emergence as modified amino-acid conjugates rather than complete disappearance [82]. In a milk–cocoa matrix, dairy proteins were similarly associated with greater flavan-3-ol stability during gastric residence, although recovery during the intestinal phase remained low (27–42%), indicating that early “protection” does not necessarily translate into high intestinal availability [83].

Evidence gaps: Release kinetics for major phenolic acids and flavonoids are described relatively often, but the outcome of complex polymeric structures (e.g., melanoidins, high-degree-of-polymerization proanthocyanidins) remains poorly resolved by LC–MS in these datasets. Many studies document disappearance of parent compounds without structurally elucidating the specific degradation products that would be necessary to sustain mechanistic continuity across digestion phases.

### 5.2. Bioaccessibility and Bioavailability: Alignment with Molecular Evidence

Bioaccessibility and bioavailability should be clearly distinguished. The former reflects solubility and presence in the intestinal lumen, whereas the latter concerns uptake and systemic circulation. Nevertheless, numerous studies show that high extraction yields or increased total phenolic content do not reliably reflect physiological exposure.

In systems that integrate cell-based transport with metabolomics, molecular size and lipophilicity behave as decisive filters. A Caco-2 transport model coupled with metabolomic profiling was used to assess phenolic bioavailability (Tier B). Phenolic transfer from digested matrices was selective: although quebracho extracts exhibited the highest total phenolic content, cellular transport was minimal, consistent with the predominance of high-molecular-weight tannins. In contrast, lower-molecular-weight phenolics from violet rice and yellow maize, including free phenolic acids and simple flavonoids, were transported more efficiently [84]. Selective transport was also observed in tiger nut beverages, where high bioaccessibility indices (up to 95%) did not translate into uniformly high Caco-2 transport, which instead favored smaller molecules such as hydroxyphenolic acids [78].

Processing can modulate this bioaccessibility filter, as it alters how tightly phenolic compounds are retained within the food matrix and how readily they are released during digestion. Germination provides a clear example. In sorghum, germination increased the bioaccessibility of 3-deoxyanthocyanidins and flavones, an effect that is consistent with cell wall loosening and the partial hydrolysis of macromolecular complexes that occurs during sprouting [85]. A similar principle applies to solvent-based processing, although the underlying mechanism differs. Natural deep eutectic solvents (NADES) improved the extraction efficiency of quince polyphenols compared with ethanol. High digestive bioaccessibility of chlorogenic acid and rutin was maintained across simulated gastrointestinal phases (Tier A) [86]. This observation indicates that solvent composition influences extraction yield. It also affects analyte stability and phase partitioning within the chyme.

Even so, the alignment between molecular evidence and outcome metrics is often imperfect. This gap is most apparent when “bioaccessibility” is inferred from proxy readouts. For carrot pomace, high bioaccessibility indices were reported using bulk spectrophotometric assays (Tier C). However, no high-resolution MS data were available to distinguish the contribution of intact polyphenols from that of degradation products or other assay-responsive constituents to the observed antioxidant signal [87]. As a result, the evidence falls short of the minimum level of alignment required when interpretations extend beyond extraction yield. LC–MS should quantify defined compounds in the intestinal filtrate. Stability versus transformation should be tested directly, for instance, by tracking marker products such as protocatechuic acid that can arise from anthocyanin breakdown. Bioavailability estimates should also rely on transport models (e.g., Caco-2), rather than cell-free dialysis, when the objective is to infer absorptive potential.

### 5.3. Colonic Microbial Biotransformation: LC–MS-Tracked Metabolites and Limitations

Polyphenols that evade small-intestinal absorption become substrates for colonic microbiota, making microbial biotransformation a central determinant of the exposure profile. In this context, LC–MS is indispensable for mapping phenolic-derived catabolites generated by gut bacteria.

Tier B untargeted metabolomics repeatedly shows that fermentation produces a chemical space that differs substantially from the parent extract. During fermentation of cocoa shell phenolics, a shift from complex flavan-3-ols toward smaller catabolites was reported, including phenyl-γ-valerolactones and phenylpropionic acids, which is relevant because parent procyanidins (e.g., procyanidin B2) are largely unabsorbable, whereas their catabolites represent more plausible exposure candidates [88]. LC–MS tracking of pomegranate peel fermentation also showed conversion of ellagic acid into urolithins (specifically urolithin A) and described distinct “metabotypes,” in which urolithin A production depended on microbial modulation involving taxa such as Bifidobacterium and Bacteroides [89].

Some studies also frame the interaction as bidirectional: polyphenols are metabolized while microbiota composition and fermentation outputs shift. Mango peel powder supplementation in yoghurt promoted *Lactobacillus* and *Bifidobacterium* while increasing SCFAs such as butyrate and propionate [90]. Olive fiber polyphenols were likewise linked with the abundance of *Faecalibacterium prausnitzii,* which is a key butyrate producer [91].

Limitations: A persistent constraint is attribution. In complex fecal fermentation systems (e.g., fecal slurries), it is often difficult to determine whether a metabolite derives specifically from microbial action on the polyphenol fraction or from parallel fermentation of other matrix components, including fibers and proteins [88,92]. Structural certainty is also limited: many metabolites are assigned only at Tier C (exact mass matches) or Tier B (MS/MS fragmentation matching databases) without confirmation by authentic standards, which are often unavailable for specialized microbial catabolites [88,89]. Finally, kinetics complicate interpretation; substantial release of bound phenolics in oat bran required 12 to 24 h of fermentation, a window in which other bioactive constituents may also change, making it difficult to isolate polyphenol-specific effects without tightly controlled designs [81].

### 5.4. Functional Outcomes: Evidence Strength and Mechanistic Attribution

Functional outcomes ranged from simple chemical antioxidant readouts to enzyme inhibition and cell-based responses. The interpretive strength depended on whether the tested fraction was clearly linked to compound-resolved LC–MS evidence.

Chemical antioxidant assays (Tier C) were reported in most studies. DPPH, ABTS, and FRAP were mainly used for screening and comparison. These assays are useful for quality control, but they may not reflect biological activity. They can respond to assay-reactive components other than intact polyphenols. For example, higher DPPH values after cooking onions were reported despite phenolic losses, which may relate to Maillard-derived products affecting the assay signal [93]. In some studies, germination and natural deep eutectic solvents (NADES) extraction were supported mainly by these chemical endpoints. This indicates antioxidant capacity rather than confirmed bioactivity in biological models [85,86].

Enzyme inhibition assays (Tier B) provided a closer link to physiological relevance. LC–MS-characterized extracts of *Allmania nodiflora* inhibited α-amylase and α-glucosidase. The activity was associated with bioaccessible flavonoids, including rutin and catechin [94]. Lipase inhibition was also tested in tiger nut beverages, and moderate inhibitory activity remained after simulated digestion [78].

More informative in vitro evidence (Tier B) came from cell-based assays coupled with molecular characterization of digested fractions. Digested honey fractions reduced nitric oxide (NO) production in LPS-stimulated RAW 264.7 macrophages, linking the bioaccessible phenolic pool to an anti-inflammatory endpoint [77]. In Caco-2 cellular antioxidant activity assays, intracellular metabolites derived from violet rice reduced oxidative stress, even when total phenolic levels were lower than in other matrices [84]. Cytotoxicity assays in HepG2 and HeLa cells were used to evaluate safety and potential anti-proliferative effects of macroalgae extracts. Reported responses were associated with compounds such as fucoxanthin and phlorotannins identified at Tier B [95].

Overall, the evidence supports a tiered interpretation of functionality. Chemical antioxidant capacity alone is a weak predictor of biological effects. Stronger functional claims were observed when LC–MS-tracked metabolites (e.g., urolithin A or defined phenolic acids) were directly linked to biological outcomes, such as NO reduction or changes in fermentation-related endpoints [77,89]. By contrast, studies reporting only TPC or DPPH after digestion, without compound-resolved confirmation of what was present in the active fraction, provide weaker support for health-relevant attribution [87].

### 5.5. Integrated Study Designs to Connect Processing, Digestion, and Function

Bridging molecular fingerprints to validated health outcomes will require study designs that integrate processing, LC–MS characterization, digestion, and functional readouts instead of treating them as separate endpoints. Based on the limitations and practices, two integrated templates can be articulated.

#### 5.5.1. Template 1: The Matrix-Protection Tracking Design

This template addresses how processing aids and matrix interactions shape not only apparent stability but also uptake and the chemical forms that persist during digestion [79,82,83]. A single polyphenol-rich extract (e.g., an anthocyanin source) would be formulated into two matrices—for example, a free solution versus a protein-bound or hydrocolloid-thickened system. LC–MS would combine targeted quantification (Tier A) of the parent compound with untargeted profiling (Tier B) to capture adducts such as protein–phenol conjugates. The system would then be tracked through a standardized static in vitro digestion model (INFOGEST), followed by Caco-2 monolayer transport, with endpoints including basolateral transport efficiency (%) and intracellular stability. The deliverable is a direct test of whether “protection” during digestion translates into reduced uptake through steric hindrance or enhanced delivery through stabilization and sustained availability.

#### 5.5.2. Template 2: The Metabolite-Function Feedback Loop

This template targets colonic biotransformation by explicitly linking time-resolved metabolite trajectories to functional markers [88,89,96]. A fiber–polyphenol co-product (e.g., fruit peel) would be subjected to non-thermal processing and then carried through digestion into an in vitro colonic fermentation model using pooled or distinct metabotype inocula. LC–MS metabolomics would be performed across multiple time points (0, 12, 24, 48 h) to annotate Tier B catabolites as they emerge, while functional endpoints, such as SCFA production and a prebiotic index, would be measured in parallel. The intended output is a defensible mapping of which microbial metabolites, rather than parent polyphenols, track most closely with the functional prebiotic effect.

**Figure 3:**
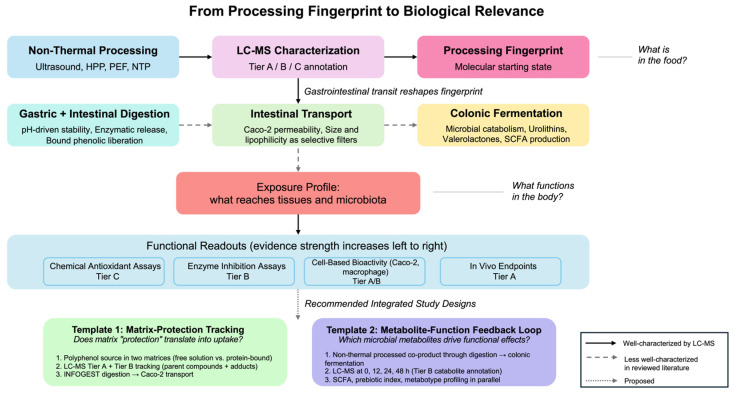
From processing fingerprints to biological relevance. The workflow links non-thermal processing and LC–MS characterization to gastrointestinal digestion outcomes (bioaccessibility and stability), intestinal transport, colonic microbial biotransformation, and functional readouts. Dashed arrows indicate steps where compound identity and transformation are currently less well characterized by LC–MS evidence in the reviewed literature.

In sum, the LC–MS molecular fingerprint is better treated as a starting state than as an endpoint: digestion and the microbiota reshape it into a different exposure profile. The reviewed literature shows that non-thermal processing and matrix formulation can influence the persistence of phenolics, but GI transit and fermentation consistently redirect them toward a more constrained set of accessible metabolites. The most biologically informative studies are those that avoid assuming “what is in the food is what functions in the body,” and instead use high-resolution analytics to follow the actual bioactive species formed in situ. Figure 3 summarizes this complete workflow from non-thermal processing and LC-MS characterization through gastrointestinal digestion, microbial biotransformation, and functional readouts, together with two proposed integrated study design templates.

## 6. Industrial Translation of LC–MS Fingerprinting for Process Optimization, Quality Assurance and Quality Control, and Standardization

Translating polyphenol analysis from bench-scale studies to industrial use depends on converting chromatographic complexity into practical process decisions. The field is moving from univariate yield metrics such as total phenolic content toward multivariate LC–MS fingerprinting with chemometrics. The main obstacles to industrial translation are analytical cost, long run times, extensive post-acquisition data processing, reliance on bench-scale datasets, limited pilot- or factory-scale validation, weak interoperability of feature tables across laboratories, and poor integration of fingerprint outputs into automated process-control systems. This section reviews evidence for applying fingerprints in process optimization, QA/QC, and standardization using a tiered evidence framework to judge implementation readiness.

### 6.1. LC–MS-Guided Process Optimization: Targets and Decision Rules

Optimization has often focused on maximizing extraction yield or total antioxidant activity. Recent studies show that LC–MS fingerprints can define workable process windows by tracking marker panels in Tier A or B rather than bulk yield alone. Markers range from single quantified analytes to multivariate patterns.

A Q-marker (quality marker) strategy is shown for *Cimicifuga* Rhizoma processing. Ultra-high-performance liquid chromatography coupled with quadrupole time-of-flight mass spectrometry (UPLC-Q-TOF/MS) was used to compare raw, wine-processed, and honey-processed materials, identifying twelve markers in Tier A or B, including isoferulic acid and ferulic acid, with strong shifts across processing routes. Processing auxiliaries such as wine and vinegar generates distinct signatures that can be targeted during optimization [97]. In hops, UPLC-Q-TOF/MS identified ten differential markers in Tier A or B, including humulone and cohumulone, supporting optimization targets matched to the intended chemotype or variety rather than a generic phenolic profile [98].

In waste-valorization work, molecular profiles often drive parameter choice. A study linked specific time–temperature settings, for example 80 °C for 60 min, to improved recovery of catechins and gallic acid from tea factory waste, and used the molecular profile to ensure harsh conditions did not erode heat-sensitive compounds [99]. In pigmented rice bran microencapsulation, LC-MS/MS tracking treated retention of cyanidin-3-glucoside and related anthocyanins as the optimization endpoint in Tier B [100].

Some works report decision rules that connect unit operations to molecular outcomes. For walnut pellicle drying, LC-MS/MS quantification against standards in Tier A showed freeze-drying best preserved monomeric phenols such as protocatechuic acid and gallic acid, while hot-air drying caused large losses. The operational rule was explicit: choose freeze-drying when retention is critical; use vacuum drying as a cost compromise; hot-air drying produced a fingerprint change judged unacceptable in that context [101]. In sesame processing across roasting and paste production, metabolomics based on ultra-high-performance liquid chromatography coupled with photodiode array detection and quadrupole time-of-flight mass spectrometry (UPLC-PDA-Q-TOF/MS) captured roasting-associated Maillard products alongside shifts in lipid-related metabolites. This implies roast time and temperature can be tuned to balance desirable flavor precursors against phenolic depletion, with fingerprints used as feedback [102].

Other workflows keep fingerprints secondary. Ultrasound-assisted extraction was optimized using response surface methodology with TPC and antioxidant assays as primary endpoints, while LC profiling mainly served as confirmation [103]. Similar yield-oriented optimization was reported using Box–Behnken-type designs, with chromatographic profiling used mainly as a support [104]. This supports yield-led optimization but offers less precision than marker-constrained processing, where the stability of specific compounds defines operating limits [97]. The same constraint-based logic is explicit in drying decisions where molecular retention is treated as non-negotiable [101].

A closed-loop logic emerges across fingerprint-led approaches. Samples are taken from intermediates such as raw material, dried products, and extract. LC–MS is acquired using full-scan or targeted MRM, then aligned and normalized, often using an internal standard. A samples-by-features matrix is summarized by heatmaps or Principal Component Analysis (PCA). Chemometrics becomes the decision node. Batches are compared to a reference fingerprint, such as a golden batch, using similarity or distance metrics, and key Q-markers are checked against tolerances. If within tolerance, processing continues or the lot is released. If not, discriminating features are localized using loadings or VIP scores and corrective actions follow the deviation pattern. Thermal load can be reduced when degradation products rise, including aglycones [97]. Marker panels can also be used to distinguish variety-linked baselines from processing effects when defining targets [98]. For roasting systems, fingerprint feedback supports balancing formation of desirable products against losses in native phenolics [102].

Even so, evidence for optimization-ready industrial use remains limited. Most studies are bench-scale and rarely demonstrate fingerprint behavior at pilot or factory volumes [104]. The same limitation is evident in broader profiling workflows that remain primarily laboratory-facing [105]. Explicit engineering thresholds are uncommon; results are usually reported as relative differences and statistical significance rather than stop criteria tied to marker concentrations [102]. This gap is also evident in workflows where molecular retention is used as the endpoint but not translated into quantified control limits [100].

### 6.2. Industrial Quality Assurance and Quality Control: Batch Consistency, Authenticity, and Marker Monitoring

LC–MS fingerprints are increasingly positioned for QA/QC, especially batch consistency, raw-material authentication, and adulteration detection. In practical deployment, raw materials are first fingerprinted to establish cultivar- and origin-specific baselines, after which a reference or golden batch is defined. Incoming lots are then analyzed together with pooled QC samples and internal-standard normalization, compared against marker tolerances and similarity metrics, and classified as accepted, released for rework, or rejected depending on whether discriminant features indicate raw-material variability, excessive thermal load, or process drift. Botanical variability nevertheless complicates industrial baselines. Sesame metabolomic profiles differed by geographic origin across Egypt, Sudan, and Nigeria, supporting a QA/QC sequence in which raw materials are fingerprinted first so intrinsic variability can be separated from processing effects at the method-validation stage [102].

Evidence is stronger for authenticity work. Berry seed phenolic profiles measured by ultra-high-performance liquid chromatography coupled with linear ion trap–Orbitrap mass spectrometry (UHPLC–LTQ Orbitrap MS) in Tier A or B provided taxonomic markers, and diagnostic flavonoid glycosides and anthocyanins distinguished *Rubus* from *Fragaria* to support mislabel detection [106]. Non-MS fingerprints also support high-throughput screening: high-performance liquid chromatography with fluorescence detection (HPLC-FLD) fingerprints combined with class-modeling detected fraud in protected designation of origin (PDO) paprika by defining an authentic class space and flagging substituted samples outside it [107]. High-performance thin-layer chromatography (HPTLC) profiling of market green tea products found that many commercial samples deviated from botanical references, supporting routine comparison to reference materials despite lower resolution in Tier C [108].

Across this literature, QA/QC implementation is best supported as offline, retrospective testing rather than online monitoring. Comprehensive profiling in Tier B or A often involves long run times and substantial post-processing, which fits release testing but not in-line control [109]. The same constraint applies to broader untargeted workflows used for compositional separation rather than real-time decisions [105]. Validation elements including the limit of detection (LOD), limit of quantification (LOQ), and recovery were reported, supporting routine use in QC laboratories at the method-validation stage [109]. Integration of fingerprint outputs into automated process-control systems is rarely shown.

Overall, fingerprints are well supported for authenticity and moderately supported for retrospective batch consistency. Chemometric models can classify samples by origin or processing type at the method-validation stage [97]. Similar classification logic supports variety-specific acceptance concepts in raw-material control [98]. However, factory-ready pass or fail rules are seldom described as operational criteria; most studies present clustering or separation rather than predefined acceptance regions that drive reject or accept decisions.

### 6.3. Standardization Roadmap: Reporting, Validation, and Data Sharing

Wider industrial uptake is constrained by fragmented reporting and limited interoperability. A practical roadmap follows from recurring strengths and gaps. Inconsistent metadata remains a major barrier to reproducibility and cross-study synthesis. More complete reporting contrasts with exploratory workflows and supports adopting a minimum checklist that preserves utility for both targeted and fingerprint-based studies [109]. Similar strengths are evident where standards-based quantification supports clearer transferability [101]. In contrast, exploratory workflows often rely on broader annotation with weaker interoperability [105]. To facilitate reproducibility and evidence-weighted synthesis across studies, Table 2 summarizes a minimum reporting checklist together with a staged standardization roadmap for LC–MS-based investigations of non-thermal processing effects on polyphenols.

At minimum, sample descriptions should state matrix conditions such as moisture and particle size and clearly report origin, because baseline fingerprints depend strongly on cultivar and provenance [102]. Variety effects in hop fingerprints highlight the same requirement for origin and cultivar metadata [98]. Extraction details should be reported with enough precision to reproduce solvent ratios, temperature, and time, since small parameter differences can shift composition [103]. The same sensitivity to parameter choice is apparent across ultrasound-assisted designs that otherwise emphasize bulk endpoints [104]. Identification confidence should be declared using tiers, separating Tier A confirmation with authentic standards from Tier B MS/MS-supported putative identification and Tier C annotation-level signals [109]. Tier A practice is also illustrated where quantification is anchored to standards [101]. Workflows with heavier reliance on putative annotations further underline the need to state confidence explicitly [105].

The quantification basis must also be explicit. Relative abundance supports within-study comparisons but limits inter-laboratory transfer compared with absolute calibration-based reporting [97]. The same limitation applies where molecular retention is tracked as an optimization endpoint without absolute scaling [100]. Standard-based quantification provides a clearer transfer pathway for industrial control [101]. Where chemometrics are used, reporting should include validation sufficient to support robustness claims [97]. Comparable class-modeling logic for fraud control also depends on transparent model reporting [107].

Data sharing is another bottleneck. Targeted HPLC studies differ from untargeted MS workflows, where reproducibility is constrained by limited shared spectral evidence [110]. Similar constraints apply where compound-focused workflows do not provide reusable feature-level data for broader re-analysis [111]. Untargeted metabolomics studies are particularly affected when feature tables and spectra are not shared [102]. This limitation is amplified in large-scale profiling, where many annotations remain putative [105]. A practical requirement is chemometric reproducibility: feature tables listing m/z, retention time, and intensity should be deposited to enable verification and re-analysis. Identifying common peaks across batches can seed shared libraries, and consistent reporting of MS/MS fragments for key Q-markers would allow verification without every laboratory purchasing every standard [97].

A three-stage pathway reflects current practice. Stage 1 reports TPC and yield supported by simple HPLC markers such as gallic acid [103]. Similar baseline approaches are seen in yield-led optimization designs [104]. Stage 2 uses untargeted LC–MS fingerprinting with multivariate visualization such as PCA to monitor consistency and identify discriminating features [102]. Comparable profiling logic is used in broader metabolomics workflows for separation and interpretation [105]. Stage 3 integrates defined Q-marker panels with absolute quantification and acceptance thresholds linked to processing parameters, aligning with optimization-led approaches [97]. The same stage benefits from Tier A quantification when targets require strict retention [101]. It also aligns with optimization framed around molecular recovery under defined time–temperature conditions [99].

In sum, LC–MS fingerprinting can distinguish varieties, track processing-driven chemical shifts, and support fraud detection, while broader industrial translation largely remains at a method-validation stage. Progress will depend on tighter reporting and greater transparency of spectral and feature-level data to improve transferability across laboratories and product systems.

## 7. Perspectives and Future Directions

The most urgent gap is lack of comparability across studies. Future work should prioritize benchmark designs that use shared matrices, technology-specific dose descriptors, pooled QC samples, and openly reported feature tables so that cross-platform synthesis is possible. This is especially important for metabolically active matrices, where sampling time and quenching practice can decide whether an observed increase is interpreted as chemistry or physiology.

The second urgent gap is the inability to distinguish release from true conversion. This requires mass-balance-oriented designs that track free, bound, and product pools within the same matrix across a controlled process gradient. Transformation language should be reserved for compound-resolved trajectories supported by interpretable MS/MS and standards where feasible, whereas multivariate separation without structural resolution should be framed more cautiously as processing-associated variation. A minimum reporting checklist is overdue for this field, including matrix state and provenance, extraction and recovery context, acquisition parameters, QC strategy, and transparent annotation confidence.

The third urgent gap is the weak link between processing fingerprints and exposure-relevant metabolites. The most defensible next step is integrated design: processing and LC–MS characterization, followed by standardized digestion, transport models where relevant, and time-resolved fermentation when colonic fate is part of the claim. These workflows make it possible to identify which processing-driven shifts persist into the bioaccessible fraction and which are analytical artifacts of extraction or matrix disruption. Among the four technologies reviewed, cold plasma warrants particular attention in this integrated agenda because its distinctive chemistry, especially oxidation, nitration potential, and surface-limited penetration, raises both efficacy and safety questions that cannot be addressed from global phenolic indices alone. On the translation side, routine industrial QA/QC will likely rest on small, robust marker panels tied to acceptance limits and golden-batch similarity metrics rather than on full untargeted fingerprints.

## 8. Conclusions

LC–MS evidence shows that non-thermal processing cannot be considered safe for the chemical structure of polyphenols. Across food matrices, ultrasound and high-pressure processing predominantly reshape phenolic fingerprints through cell disruption and repartitioning, so apparent post-treatment increases often reflect improved extractability from bound pools rather than de novo formation. Pulsed electric fields follow the same release logic via electroporation, but outcomes can shift toward oxidative loss when membrane rupture increases contact with endogenous oxidases or when subsequent steps amplify degradation. Non-thermal plasma stands apart by generating reactive oxygen and nitrogen species that can drive surface-level oxidation and nitration; in fresh-cut tissues, similar stress can also stimulate phenylpropanoid metabolism, producing fingerprints that blend abiotic chemistry with biological response. The LC–MS evidence framework does not eliminate all uncertainty, but it materially improves the distinction between apparent change and true transformation. Within this framework, unchanged or higher signals for constitutive compounds are interpreted as preservation or enhanced extractability unless identifiable products or adducts support structural conversion. True transformation is therefore reserved for compound-resolved cases, especially oxidation or nitration products, where identification confidence is explicit. Practically, adopting small marker panels that capture release, sensitivity, and technology-specific conversion and tracking them through digestion and microbial metabolism will better align process selection with functional relevance and support transferable industrial QA/QC.

## Figures and Tables

**Table 1:** Evidence inventory of representative LC–MS studies underpinning the technology-specific synthesis of non-thermal processing effects on polyphenol profiles, with dominant evidence-tier assignments.

Technology	Matrix/Food System	Analytical Platform	Provisional Dominant Tier	Phenolic Evidence Type	Archetype Supported	Reference
HPP	Blueberry juice	HPLC-MS/MS (WTM)	B	Compound Profile & Quant	Stabilization; no Maillard products	[45]
HPP	Spine grape juice (NFC)	HPLC-MS/MS	B	Compound Profile	Stabilization; similar to fresh	[46]
HPP	Blended fruit–veg juice	UPLC-QTOF-MS	B/C	Untargeted Profile	Release; minor isomerization	[47]
HPP	Tomato juice	UPLC-QTOF-MS	B/C	Compound Profile	Release (increased extractability)	[48]
HPP	Apple juice (cloudy)	LC-MS/MS (Targeted)	A/B	Compound Profile	Release (Procyanidins/Phloretin)	[49]
HPP	Aronia juice	HPLC-MS/MS	B	Compound Profile	Stabilization vs. Thermal	[50]
Ultrasound	Giloy leaves	LC-QTOF-MS/MS	B	Compound Profile	Release (Extraction optimization)	[51]
Ultrasound	Blackthorn fruit	LC-MS/MS	B	Compound Profile	Release	[52]
Ultrasound	Strawberry tree fruit	LC-MS/MS	B	Compound Profile	Release	[53]
Ultrasound	Wheat bran	LC-MS	B/C	Compound Profile	Release (Bound phenolics)	[54]
PEF	Prunes (during drying)	HPLC-MS/MS (WTM)	B	Compound Profile	Degradation/Oxidation	[55]
PEF	Pineapple flesh	UPLC-MS/MS	B	Compound Profile	Release	[56]
PEF	Broccoli by-products	UHPLC-QTOF-HRMS	B/C	Compound Profile	Release; metabolic signature	[57]
PEF	Orange peel	UPLC-IMS-MS	B/C	Compound Profile	Release	[58]
PEF	Ziziphus lotus	HPLC-DAD-ESI-MS	B/C	Compound Profile	Release	[59]
NTP	Apple slices	HPLC-QTOF-MS	B/C	Compound Profile	Structural conversion (oxidative)	[44]
NTP	Model systems (Phenolic)	HPLC-DAD; LC-MS	B/C	Compound Profile	Chemical transformation (Nitration)	[43]
NTP	Strawberries (Fresh cut)	GC-MS (primary)/TPC	C	Profile & Total Index	Metabolic stress response	[60]
NTP	Lamb’s lettuce	HPLC-DAD; LC-MS	B/C	Compound Profile	Oxidation (Quercetin)	[61]
NTP	Blue pea flower	UPLC-ESI-MS/MS	B	Compound Profile	Release vs. Degradation	[62]
NTP	Rocket salad (PAW)	HPLC-DAD-MS/MS	B	Compound Profile	Retention/Minor degradation	[63]

**Table 2:** Minimum reporting checklist and staged standardization roadmap for LC–MS-based studies on non-thermal processing effects on polyphenols.

**Panel A. Minimum reporting checklist**			
**Category**	**Minimum required reporting**		**Why it matters for synthesis**
Sample and matrix metadata	Cultivar/variety, maturity, moisture content, particle size, and pre-treatment handling		Matrix heterogeneity strongly affects extractability and apparent processing effects
Processing parameters	Technology-specific dose descriptors (e.g., ultrasound power density; pressure level; PEF field strength and pulse width; plasma power/voltage, gas composition, exposure time); record treatment duration		Enables cross-study comparison beyond nominal processing labels
Temperature and environment	Temperature profile during treatment; headspace or oxygen exposure when relevant		Distinguishes non-thermal effects from secondary thermal or oxidative contributions
Extraction and recovery	Solvent system, extraction protocol, and recovery assessment; use of internal standards where feasible		Prevents conflation of extractability with true chemical transformation
LC–MS acquisition	Ionization mode, mass analyzer, mass resolution, MS/MS acquisition strategy		Determines identification confidence and comparability of detected features
Quality control (QC)	Pooled QC samples, blanks, randomization order, and drift monitoring		Ensures analytical robustness and reduces false-positive variation
Annotation confidence	Explicit declaration of identification tier (Tier A/B/C) for reported compounds or features		Aligns mechanistic claims with evidential strength
Quantification basis	Absolute calibration, semi-quantitative, or relative abundance clearly stated		Prevents overinterpretation of fold-changes as concentration changes
Data availability	Feature tables including *m*/*z*, retention time (RT), and intensity; MS/MS spectra for key discriminants		Enables re-analysis, meta-synthesis, and method transfer
**Panel B. Staged standardization roadmap**			
**Stage**	**Primary goal**	**Typical outputs**	**What it enables**
Stage 1: Baseline screening	Rapid assessment of gross processing effects	Total phenolics, simple HPLC markers, yield changes	Preliminary trend screening (not sufficient for transformation claims)
Stage 2: Fingerprinting and discrimination	Characterization of processing-associated chemical variation	Untargeted LC–MS fingerprints, multivariate visualization (e.g., PCA), discriminant features	Technology comparison and hypothesis generation
Stage 3: Compound-resolved validation	Establishment of robust, transferable markers	Tier A-validated compounds, absolute quantification, defined marker panels	Mechanistic interpretation, QA/QC benchmarks, and industrial implementation

Footnote: Identification confidence tiers (Tier A–C) follow the LC–MS evidence framework defined in Section 2. Feature tables should minimally include *m*/*z*, retention time (RT), and intensity; MS/MS spectra should be provided for key discriminant features where applicable.

## Data Availability

No new data were created or analyzed in this study. Data sharing is not applicable to this article.

## Abbreviations

The following abbreviations are used in this manuscript:

ABTS	2,2′-azino-bis(3-ethylbenzothiazoline-6-sulfonic acid)
AI	Artificial intelligence
CO_2_	Carbon dioxide
DBD	Dielectric barrier discharge
DPPH	2,2-diphenyl-1-picrylhydrazyl
FRAP	Ferric reducing antioxidant power
GC–MS	Gas chromatography–mass spectrometry
GI	Gastrointestinal
HPLC	High-performance liquid chromatography
HPLC-FLD	High-performance liquid chromatography with fluorescence detection
HPP	High-pressure processing (high hydrostatic pressure processing)
HPTLC	High-performance thin-layer chromatography
HRMS	High-resolution mass spectrometry
HTST	High-temperature short-time
INFOGEST	INFOGEST standardized in vitro digestion protocol
LC	Liquid chromatography
LC–MS	Liquid chromatography–mass spectrometry
LC–MS/MS	Liquid chromatography–tandem mass spectrometry
LOD	Limit of detection
LOQ	Limit of quantification
LPS	Lipopolysaccharide
MRM	Multiple reaction monitoring
MS	Mass spectrometry
MS/MS	Tandem mass spectrometry
NADESs	Natural deep eutectic solvents
NFC	Not from concentrate
NO	Nitric oxide
NTP	Non-thermal plasma
PAW	Plasma-activated water
PCA	Principal component analysis
PDO	Protected designation of origin
PEFs	Pulsed electric fields
POD	Peroxidase
PPA	Plasma processed air
PPO	Polyphenol oxidase
QA/QC	Quality assurance/quality control
QC	Quality control
RONS	Reactive oxygen and nitrogen species
RSM	Response surface methodology
RT	Retention time
SCFAs	Short-chain fatty acid(s)
TFC	Total flavonoid content
TPC	Total phenolic content
UAE	Ultrasound-assisted extraction
UHPLC	Ultra-high-performance liquid chromatography
UHPLC-MS/MS	Ultra-high-performance liquid chromatography–tandem mass spectrometry
UPLC	Ultra-performance liquid chromatography
UPLC-ESI/MS/MS	Ultra-performance liquid chromatography–electrospray ionization–tandem mass spectrometry
UPLC-IMS-MS	Ultra-performance liquid chromatography–ion mobility spectrometry–mass spectrometry
UPLC-MS/MS	Ultra-performance liquid chromatography–tandem mass spectrometry
UPLC-PDA-Q-TOF/MS	Ultra-performance liquid chromatography–photodiode array–quadrupole time-of-flight mass spectrometry
UPLC-Q-TOF/MS	Ultra-performance liquid chromatography–quadrupole time-of-flight mass spectrometry
UHT	Ultra-high temperature
UV	Ultraviolet
UV-C	Ultraviolet C
VIP	Variable importance in projection
WTM	Widely targeted metabolomics
·OH	Hydroxyl radical
